# Hybrid NiMn‐LDHs as Active Materials for Electro‐Oxidation of Organic Pollutants

**DOI:** 10.1002/gch2.70155

**Published:** 2026-09-25

**Authors:** Keyvan Mirehbar, Javier Llorente‐López, Alvaro Seijas‐Da Silva, Camilo Jaramillo‐Hernández, Petra Batinić, Paula Porawski, Miguel García‐Tecedor, Víctor A. de la Peña O'Shea, Jesús Palma, Gonzalo Abellán, Julio J. Lado

**Affiliations:** ^1^ Electrochemical Processes Unit IMDEA Energy Institute Móstoles, Madrid Spain; ^2^ Escuela de Doctorado UAM, Centro de Estudios de Posgrado Universidad Autónoma de Madrid, Ciudad Universitaria de Cantoblanco Madrid Spain; ^3^ Photoactivated Processes Unit IMDEA Energy Institute Móstoles, Madrid Spain; ^4^ Institute of Molecular Science (ICMol) University of Valencia (UVEG) Paterna Spain; ^5^ Matteco Team, S.L., Carrer de Les Noves Tecnologies Paterna, Valencia Spain

**Keywords:** carbon nanotubes (CNT), CO_2_ reduction reaction (CO_2_RR), electrochemical oxidation, hybrid electrochemical systems, hydroxyl radicals, NiMn layered double hydroxides (LDH), phenol degradation, wastewater treatment

## Abstract

Electrochemical oxidation of phenol is a promising route for sustainable treatment of wastewater containing toxic aromatic pollutants, but its practical application is limited by sluggish kinetics and competing anodic side reactions. Here, we report NiMn‐based layered double hydroxide electrocatalysts (NiMn‐LDH) that enhance phenol degradation under mild electrochemical conditions. Mn incorporation into the LDH structure provides accessible higher oxidation states, improving redox flexibility, promoting hydroxyl radical (·OH) generation, and suppressing the competing oxygen evolution reaction. Three variants, NiMn‐LDH‐CNT, NiMn‐LDH‐CMC, and NiMn‐LDH‐Tris, were synthesized to assess the influence of conductive additives and synthesis media. The CNT‐supported catalyst showed the highest activity, achieving >95% phenol removal within minutes at current densities up to 20 mA cm^−2^, while maintaining a nearly constant cell voltage over approximately 200 h of operation. High removal efficiencies, ca. 90%, were retained even when the Na_2_SO_4_ concentration was decreased from 100 to 1 mM and under synthetic wastewater conditions, confirming system robustness. The optimized NiMn‐LDH electrocatalyst was further integrated as the anode in a hybrid cell coupling phenol oxidation with CO_2_ reduction at a Cu/CuO cathode, enabling simultaneous wastewater remediation and CO_2_ conversion to value‐added hydrocarbons.

## Graphical abstract

NiMn‐LDH electrocatalysts enable rapid phenol degradation by combining enhanced redox flexibility with efficient reactive oxygen species generation. Integration of the optimized anode with a Cu/CuO cathode simultaneously removes organic pollutants from wastewater and converts CO_2_ into value‐added hydrocarbons, demonstrating an efficient hybrid strategy for environmental remediation and carbon utilization.

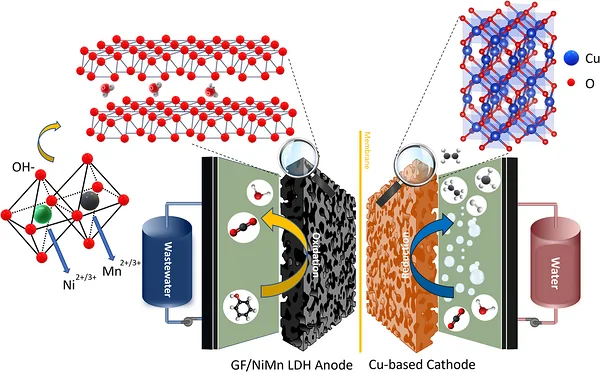

## 1 Introduction

Industrial wastewater streams frequently contain phenolic compounds, which pose significant environmental risks due to their toxicity, persistence, and resistance to conventional treatment methods [1, 2]. At the European level, phenol is recognized as a priority hazardous pollutant because of its stability and widespread occurrence in industrial effluents such as those from petrochemical, pharmaceutical, and resin production processes [3, 4]. Indeed, many Best Available Techniques (BAT) reference documents under the European Industrial Emissions Directive (IED) establish stringent limits for phenolic compounds in industrial effluents. For instance, the BAT Conclusions for the Refining of Mineral Oil and Gas (EU 2014/738/EU) [5] specify that emissions of phenols to water should generally be maintained at ≤0.5–1 mg L^−1^, depending on plant configuration and treatment schemes. Similarly, the BAT Reference Document (BREF) for Large Volume Organic Chemicals (LVOC) and the Common Waste Water and Waste Gas Treatment/Management Systems in the Chemical Sector (CWW BREF, 2016) emphasize phenol as a key parameter to be controlled, with BAT‐AEL ranges for phenolic compounds typically below 1 mg L^−^^1^ when advanced treatment (e.g., biological treatment combined with polishing steps such as adsorption or oxidation) is implemented. Despite significant progress in emission control, its continuous release from industrial and municipal wastewater streams still raises environmental concerns, particularly at localized scales and in cases of insufficient treatment [4]. Conventional treatment techniques, including adsorption and biological degradation, often fail to achieve complete mineralization, highlighting the need for more efficient and robust treatment strategies [4]. Among the available alternatives, electrochemical oxidation has emerged as a promising approach because it enables high pollutant removal efficiencies with minimal chemical inputs and allows for on‐site treatment [6, 7]. In particular, anodic oxidation processes based on the generation of hydroxyl radicals (·OH) have been identified as highly effective for the degradation of refractory organic compounds [8]. The highly reactive ·OH species generated at the electrode surface can non‐selectively oxidize organic molecules and, under sufficiently oxidative conditions, may ultimately lead to their mineralization into CO_2_ and water [8, 9].

Among non‐noble metal electrocatalysts investigated for anodic oxidation processes, nickel‐based oxides and hydroxides have attracted considerable attention due to their low cost, high stability, and favorable redox properties [10]. These materials have been extensively studied in the context of the oxygen evolution reaction (OER), where their ability to undergo rapid redox transitions facilitates efficient charge transfer [11]. In alkaline environments, Ni(OH)_2_ can be reversibly oxidized to NiOOH, a highly active phase that plays a central role in anodic oxidation reactions [12]. The redox cycling between Ni^2+^/Ni^3+^ (and occasionally Ni^3+^/Ni^4+^) provides accessible oxidation sites that promote electron transfer and contribute to the generation of reactive oxygen species such as hydroxyl radicals (·OH) [13]. These reactive intermediates enable the effective degradation of organic pollutants. Consequently, Ni‐based catalysts have emerged as promising candidates for the electrochemical oxidation of refractory organic compounds, including phenol [9, 14]. This renewed interest also stems from the potential to selectively convert phenol into value‐added products such as benzoquinone, a compound of significant relevance to the chemical manufacturing industry [15, 16]. Beyond their application in pollutant removal, organic electro‐oxidation reactions are increasingly being investigated as alternatives to the kinetically demanding OER in paired electrolysis systems. For example, phenol oxidation has been coupled with cathodic hydrogen production to enable the simultaneous formation of benzoquinone and H_2_ [15]. Similarly, Hu et al. integrated methanol electro‐oxidation with hydrogen generation using a manganese selenide/cobalt selenide heterostructure electrocatalyst [17]. These studies illustrate how replacing conventional anodic water oxidation with an organic oxidation reaction can combine anodic treatment or chemical valorization with the generation of a useful cathodic product. Extending this concept to wastewater remediation and carbon utilization, the present work investigates phenol degradation as the anodic reaction coupled with cathodic CO_2_ reduction.

The fundamentals of proton and electron transfer processes in the electrooxidation of primary alcohols and aldehyde‐containing organic molecules on Ni(OH)_2_ catalysts in alkaline media have been previously explored [12]. The general consensus is that the electro‐deprotonated Ni hydroxide catalyst is the active phase, serving as a proton and electron acceptor in a spontaneous oxidation reaction, after which it is reduced back to its original form. A high intrinsic deprotonation propensity in the catalyst is thus desirable, as it provides abundant active sites for organic oxidation while mitigating OER [18]. In the case of phenol oxidation, optimizing the deprotonation capacity of Ni‐based catalysts could enhance the efficiency of radical‐mediated degradation pathways.

Layered double hydroxides (LDHs) have emerged as a versatile class of catalytic materials due to their tunable electronic structure, high density of accessible active sites, large specific surface area, and favorable electron transfer properties [19, 20, 21]. Among LDH systems, NiFe‐LDHs have been extensively investigated for anodic oxidation reactions, particularly for the oxygen evolution reaction, owing to their high catalytic activity in alkaline media [22, 23]. In contrast, NiMn‐based systems have received comparatively less attention in electrocatalysis despite their rich redox chemistry and the multiple accessible oxidation states of manganese [24, 25]. Previous studies have demonstrated that the synergistic combination of Ni and Mn can significantly enhance electrochemical oxidation processes. In particular, Ni─Mn binary oxide electrodes have shown superior activity toward the electrochemical degradation of phenolic pollutants compared to their single‐metal counterparts, attributed to the improved generation of reactive oxygen species and enhanced electron transfer properties [26]. Furthermore, operando spectroscopic investigations of NiMn‐based materials have revealed that the coexistence of Ni and Mn active centers facilitates the formation of highly reactive oxygen species and promotes efficient degradation pathways for organic pollutants [27]. More recently, NiMn‐LDH systems have also demonstrated promising performance in organic electro‐oxidation reactions. For example, ultrathin NiMn‐LDHs have been reported to achieve nearly 100% Faradaic efficiency for the oxidation of biomass‐derived 5‐hydroxymethylfurfural, significantly outperforming NiFe‐LDHs due to the higher deprotonation capability of Mn─OH sites and the presence of dual Ni─O and Mn─O active centers [18]. These findings suggest that the synergistic interaction between Ni and Mn can enhance proton‐coupled electron transfer processes while suppressing competing OER reactions. Building upon these insights, NiMn‐LDHs may offer a promising platform for the electrochemical oxidation of organic pollutants in wastewater treatment applications. However, despite the promising catalytic characteristics of Ni─Mn systems, their application in electrochemical advanced oxidation processes for pollutant degradation remains largely unexplored.

The selection of NiMn‐LDH in the present work was motivated by the different catalytic requirements of organic electro‐oxidation and conventional water oxidation. Although NiFe‐LDH is among the most active and widely studied non‐noble‐metal catalysts for OER, this reaction competes with the desired oxidation of organic contaminants for the applied current and surface intermediates. Consequently, maximizing OER activity does not necessarily provide the most effective anode for pollutant degradation. Replacing Fe with Mn introduces multiple accessible Mn oxidation states and complementary Ni─O and Mn─O sites, increasing the redox flexibility of the LDH structure and potentially facilitating proton‐coupled electron transfer and the formation of reactive oxygen species [18, 24, 25, 26, 27]. Mn‐containing Ni‐based LDHs have also shown a high deprotonation propensity during organic electro‐oxidation, which can favor substrate oxidation relative to competing OER pathways [18]. In the present study, the relevance of this selection is supported by a direct comparison under identical operating conditions. Under these conditions, GF/NiMn‐LDH achieved nearly complete phenol removal within approximately 20–30 min, whereas GF/NiFe‐LDH reached approximately 70%–80% removal after more than 40 min. This direct comparison supports the selection of Mn as the secondary metal and demonstrates the faster phenol‐oxidation performance of the NiMn‐LDH system.

Our previous studies established the potential of NiMn‐based mixed oxides for electrochemical wastewater treatment. A three‐dimensional NiMnO_3_ electrode exhibited enhanced phenol oxidation relative to its single‐metal counterparts, while the subsequent integration of NiMnO_3_ with reduced graphene oxide improved charge transfer and enabled operando investigation of the relationship between the catalyst's oxygen‐evolution chemistry and pollutant oxidation [26, 27]. Nevertheless, those studies focused on mixed‐oxide phases and did not examine layered double hydroxides or the influence of different LDH–carbon and LDH–organic interfaces on pollutant electro‐oxidation. Furthermore, the operation of NiMn‐based electrodes under low‐conductivity and multicomponent wastewater conditions and their integration with a value‐generating cathodic reaction remained insufficiently explored.

Herein, we address these knowledge gaps by synthesizing and systematically comparing three hybrid NiMn‐LDH materials prepared through CNT‐, CMC‐, and Tris‐assisted routes. Their structural properties, surface chemistry, morphology, electrochemical behavior, phenol‐removal performance, and operational stability are evaluated to establish relationships between material architecture and catalytic performance. A direct comparison with NiFe‐LDH and the previously reported NiMnO_3_–rGO electrode is also conducted under comparable experimental conditions. Among the investigated materials, NiMn‐LDH‐CNT combines an open nanosheet architecture, a relatively high specific surface area, and favorable charge‐transfer characteristics, resulting in the fastest phenol‐removal kinetics.

Beyond catalyst development, the optimized NiMn‐LDH‐CNT electrode is evaluated at progressively lower Na_2_SO_4_ concentrations and in a multicomponent synthetic wastewater matrix to assess its robustness under conditions more representative of practical water treatment. Finally, the electrode is integrated as the anode in a proof‐of‐concept hybrid system coupling phenol oxidation with CO_2_ reduction at a Cu/CuO cathode. Thus, the principal novelty of this work lies in introducing structurally tunable hybrid NiMn‐LDHs for electrochemical pollutant removal, establishing material–performance relationships across three LDH architectures, and demonstrating their applicability in both challenging water matrices and an integrated wastewater‐treatment and carbon‐utilization configuration.

## 2 Experimental Section

### 2.1 Synthesis of Different NiMn‐LDH (CNT, CMC, Tris)

#### 2.1.1 Synthesis of NiMn‐LDH‐CNT and NiMn‐LDH‐CMC

NiMn‐LDH was synthesized via an alkoxide‐based precipitation method [28, 29, 30]. A 40 mM metal precursor solution (30 mM Ni and 10 mM Mn) was prepared in methanol, while a 1 M NaOH solution was separately prepared in methanol as the precipitating agent. The NaOH solution was gradually added to the metal precursor solution over a period of 2–3 min under continuous stirring. The resulting mixture was stirred for 48 h at room temperature to allow for complete precipitation and crystallization of the layered double hydroxide structure.

Incorporation of carbon nanotubes (CNTs) was performed using two different types: Tuball CNTs stabilized with carboxymethyl cellulose (CMC) and commercially available CNTs (Sigma–Aldrich). Prior to use, both types underwent oxidation treatment in a microwave furnace. The CNTs were mixed with a 60 g L^−1^ hydrogen peroxide (H_2_O_2_) solution and subjected to microwave‐assisted oxidation. Following oxidation, the CNTs were filtered, washed thoroughly with deionized water, and dried under vacuum at 60°C.

For the preparation of NiMn‐LDH‐CNT composites, the oxidized CNTs were dispersed into the metal precursor solution before the addition of NaOH, allowing interaction between the CNTs and metal precursors for a duration of 1 to 24 h. Subsequently, the NaOH solution was added dropwise over 2–3 min, and the reaction mixture was stirred for 48 h. The metal‐to‐CNT ratio was maintained at 7:1. The resulting precipitate was collected, washed multiple times with ethanol and deionized water, and dried under vacuum at 60°C for 24 h.

#### 2.1.2 Synthesis of NiMn‐LDH‐Tris

NiMn‐LDH‐Tris was synthesized using a modified hydrothermal method, adapted from the protocol [31, 32]. The chloride salts of nickel and manganese (NiCl_2_·6H_2_O and MnCl_2_·4H_2_O) were dissolved in 80 mL of Milli‐Q water along with 0.5 M tris(hydroxymethyl) aminomethane (Tris) to obtain a total metal cation concentration of 0.05 M, maintaining a Ni/Mn molar ratio of 3:1.

The pH of the solution was adjusted to pH = 12 by gradual addition of a 1 M NaOH solution under stirring. The resulting suspension was mixed thoroughly and transferred to a Teflon‐lined stainless‐steel autoclave. The autoclave was placed in a preheated oven at 180°C for 24 h to facilitate hydrothermal crystallization and generate covalently functionalized LDH. After completion of the reaction, the autoclave was allowed to cool naturally to room temperature. The obtained brown precipitate was collected by centrifugation, washed multiple times with deionized water and ethanol, and dried under vacuum at 60°C for 24 h.

### 2.2 Microstructural Characterization

The crystalline structure of the NiMn‐LDH samples was analyzed using X‐ray diffraction (XRD) on a PANalytical Empyrean diffractometer equipped with Cu Kα radiation (λ = 1.5406 Å). The measurements were conducted at a scan rate of 0.2° s^−1^, with an operating voltage of 45 kV and an emission current of 40 mA. The diffraction patterns were analyzed to determine the phase composition, crystallite size, and interlayer spacing of the LDH structures. The specific surface area and pore size distribution of the samples were determined using nitrogen (N_2_) adsorption‐desorption isotherms recorded on a Quantachrome QuadraSorb‐S system. The Brunauer‐Emmett‐Teller (BET) method was applied to calculate the specific surface area. Prior to analysis, all samples were degassed under vacuum at 120°C for 12 h to remove adsorbed moisture and contaminants. Raman spectra were collected to investigate the structural integrity, bonding environment, and presence of active sites in the NiMn‐LDH samples. Measurements were performed using a Horiba LabRAM HR evolution, employing a green laser (532 nm) in the 750–3500 cm^−1^ range. A 50x Objective with a 600 mm^−1^ grating was employed to acquire all Raman spectra. Measurements were performed at least 25 times at 1.25 mm of laser power, with an acquisition time of 10 s. The chemical composition and oxidation states of Ni, Mn, O, and C in the NiMn‐LDH samples were analyzed by X‐ray photoelectron spectroscopy (XPS) using a Thermo Scientific K‐Alpha X‐ray Photoelectron Spectrometer. Al Kα X‐ray radiation was employed as an X‐ray source (1486.6 eV). For all elements, more than 100 spectra were recorded employing a step of 0.1 eV with a focused spot higher than 400 µm. XPS data were analyzed with Thermo Advantage v5.9912 software. All binding energies were referenced to the C 1s peak at 284.8 eV. The morphology and surface characteristics of the NiMn‐LDH samples were examined using scanning electron microscopy (SEM) on a JEOL JSM‐7800F Prime microscope operated at an acceleration voltage of 15 kV. The microstructure and nanoscale morphology of NiMn‐LDH were further analyzed using transmission electron microscopy (TEM). TEM images were acquired using a Philips Tecnai 20 microscope, operating with a BaF_6_ filament at an acceleration voltage of 200 kV.

### 2.3 Electrochemical Experiments

#### 2.3.1 Preparation of Electrodes

Graphite felt substrates (20 mm × 20 mm × 46 mm) were used as the anode support material. Prior to coating, the substrates were calcinated at 400°C for 4 h to enhance surface wettability.

The catalyst ink for electrode coating was prepared by dispersing 85 wt.% of the active material (1.275 g), 10 wt.% carbon black as a conductive additive (KetjenBlack EC‐600 JD, was purchased from Nanografi, MTI corporation, 150 mg), and 5 wt.% polyvinylidene fluoride (PVDF purchased from Nanografi, MTI corporation, 150 mg) as a binder in N‐methyl‐2‐pyrrolidone (NMP; ≥99% purchased from Sigma‐Aldrich.) under continuous stirring until a homogeneous colloidal suspension was obtained. The cleaned graphite felt substrates were immersed in the catalyst ink and subjected to ultrasonication for 30 min to ensure uniform deposition of the active material. The coated electrodes were subsequently dried at 80°C for 12 h under vacuum. The mass loading of the deposited active material was consistently maintained at 200 ± 5 mg across all electrode samples to ensure comparability in electrochemical performance.

#### 2.3.2 Electrochemical Characterization and Electro‐Oxidation Process in Flow Cell

A custom‐designed 3D‐printed electrochemical flow cell, previously described in our earlier work, was used for electrochemical oxidation experiments. The reactor was fabricated using UV‐sensitive resin (ANYCUBIC) and featured a flow‐through configuration, where the electrolyte flow was perpendicular to the electric current between the anode and cathode. This design was optimized to achieve uniform current and potential distribution across the electrode surface, minimize ohmic resistance, and enhance mass transport of electroactive species, thereby reducing concentration polarization effects [33, 34].

The electrochemical cell consisted of a graphite plate (Sigracell TF6) serving as the anodic current collector, positioned within the anolyte compartment, and a thin stainless‐steel plate (0.2 mm thickness, Goodfellow) acting as the cathode. A Nafion 117 membrane (Ion Power) was used to separate the anolyte and catholyte compartments, preventing cross‐contamination while allowing selective ion exchange. The electrolyte flow was maintained using a Masterflex L/S peristaltic pump, ensuring a stable flow rate. The interelectrode gap was set at 4 mm to optimize charge transfer dynamics.

The electrochemical performance of the LDH materials was evaluated using a Bio‐Logic VMP3 potentiostat controlled by EC‐Lab software in a three‐electrode configuration. Working electrodes were prepared by drop‐casting 5 µL of the corresponding NiMn‐LDH ink onto a glassy carbon substrate (area of 7.065 mm^2^). The ink was formulated following the proportions described above using 2 mL of NMP. A Ag/AgCl (3 M NaCl) electrode and a platinum mesh were employed as reference and counter electrodes, respectively. Linear sweep voltammetry (LSV) measurements were conducted in 0.1 mol L^−^^1^ Na_2_SO_4_ electrolyte at a scan rate of 1 mV s^−1^ over a potential window of 0.0–1.5 V. Cyclic voltammetry (CV) were performed in the same electrolyte within the same potential range at varying scan rates (1–100 mV s^−1^).

Electrochemical characterization was further conducted in a flow cell configuration using GF/NiMn‐LDH as working electrode and stainless steel serving as both the counter and reference electrode. In this setup, LSV´s and CV´s measurements were performed in 0.1 mol L^−1^ Na_2_SO_4_ solution at a scan rate of 10 mV s^−1^. Additional CV measurements for double‐layer‐capacitance analysis were recorded in the flow‐cell configuration at scan rates of 20, 50, 100, and 200 mV s^−1^, using the stainless‐steel electrode as a quasi‐reference. Material‐specific non‐Faradaic potential windows were selected for NiMn‐LDH‐CNT, NiMn‐LDH‐CMC, and NiMn‐LDH‐Tris. The capacitive current‐density difference was calculated as:

$$\Delta j=j_{a}-j_{c}$$

where *j_a_* and *j_c_* are the anodic and cathodic current densities, respectively, measured at the central potential of the corresponding non‐Faradaic window. The areal double‐layer capacitance was obtained from:

$$\;C_{dl}=\frac{d\left(\Delta j/2\right)}{d\nu }\;$$

where ν is the scan rate. For comparative estimation of the electrochemically active surface area, a conventional specific capacitance of *C_s_* =  40 µF cm^−2^was assumed. Because *C*_dl_ was determined from current densities normalized to the projected geometric electrode area, the roughness factor (*RF*) and apparent ECSA were calculated according to:

$$RF=\frac{C_{\mathrm{dl}}}{C_{s}}$$

$$ECSA=RF\times A_{\mathrm{geo}}=\frac{C_{\mathrm{dl}}A_{\mathrm{geo}}}{C_{s}}$$

where *A*_geo_ =  4.00cm^2^, corresponding to the projected area of the 20 mm × 20 mm electrode. Because *C_s_* is dependent on electrode composition, surface state, electrolyte, and applied potential, the calculated ECSA values are considered comparative estimates rather than absolute surface areas.

Electrochemical Impedance Spectroscopy (EIS) test was carried out in the same electrolyte, over a frequency range of 100 kHz to 100 mHz and a disturbance amplitude of 5 mV. Long‐term operational stability was evaluated by chronopotentiometry for approximately 200 h under an applied current density of 20 mA cm^−2^, using 0.1 M Na_2_SO_4_. The evolution of the full‐cell voltage was monitored as an indicator of electrochemical performance during prolonged operation. No post‐operation structural, morphological, compositional, or metal‐leaching analyses were performed after the durability test.

Electrochemical oxidation of phenol was conducted mainly in a 0.1 M Na_2_SO_4_ (Sigma‐Aldrich) solution as the supporting electrolyte. Additionally, experiments were also performed to test the effect of the supporting electrolyte in 0.01 M, 0.001 M Na_2_SO_4_ and synthetic wastewater (composition detailed in Table S1), thereby providing a closer approximation to realistic water matrices. The anode materials evaluated in this study included GF/NiMnO_3_‐rGO, GF/NiMnLDH‐CMC, GF/NiMnLDH‐CNT, and GF/NiMnLDH‐Tris, all prepared using the method described in Section 2.3.1. Each of these electrodes was consistently paired with a stainless‐steel cathode. The electrochemical experiments were conducted with an initial phenol concentration of 100 mg L^−1^ (Sigma‐Aldrich), an anolyte and catholyte volume of 100 mL each, a flow rate of 30 mL min^−1^ controlled by the peristaltic pump, and a solution pH of approximately 6.0, corresponding to the natural pH of the treated phenol solution. The flow‐through reactor configuration was designed to maximize mass transfer efficiency and provide a scalable approach for electrochemical wastewater treatment.

**TABLE 1:** Comparison of representative electrode systems for electrochemical phenol oxidation.

Anode	Phenol	Electrolyte	Operating condition	Phenol removal	Refs.
GF/NiMn‐LDH‐CNT	100 mg L^−1^	0.1 M Na_2_SO_4_; pH ≈ 6	5 mA cm^−2^; 30 mL min^−1^	>95% in approximately 30 min	This work
GF/NiMn‐LDH‐CMC	100 mg L^−1^	0.1 M Na_2_SO_4_; pH ≈ 6	5 mA cm^−^^2^; 30 mL min^−^^1^	≈100% in approximately 60–70 min	This work
GF/NiMn‐LDH‐Tris	100 mg L^−1^	0.1 M Na_2_SO_4_; pH ≈ 6	5 mA cm^−^^2^; 30 mL min^−^^1^	≈100% in approximately 60–70 min	This work
NF/NiMnO_3_	100 mg L^−1^	0.1 M Na_2_SO_4_; pH 6	Optimized galvanostatic condition	100% in approximately 60 min	[26]
GF/NiMnO_3_–rGO	100 mg L^−1^	0.1 M Na_2_SO_4_; pH ≈ 6	Galvanostatic operation	100% in approximately 30 min	[27]
SnO_2_–Sb_2_O_3_/GAC particle electrode	100 mg L^−1^	0.075 M Na_2_SO_4_; pH 3	15 V; 30 g L^−^^1^ particle loading	99.65% after 180 min	[42]

#### 2.3.3 HER/CO2RR Coupled to Phenol Oxidation Experiments

Commercial copper foam (Xiamen Lith Machine) pieces (20 mm x 20 mm x 6 mm) were calcined at 500°C for 12 h with a 10°C min^−1^ ramp to form Cu/CuO cathodes. The cathodes were used as prepared with no further treatment. The same experimental setup was employed for these experiments. HER/CO_2_RR was performed in 100 mL of CO_2_‐saturated KHCO_3_ (99.7%, Sigma‐Aldrich) 0.5 M. The CO_2_ (Nippon Gases) flow was adjusted to 50 mL min^−1^. The cathode materials evaluated in this study included commercial Cu foam and Cu/CuO foam prepared by calcination.

The volume of the anolyte was increased from 100 mL to 1 L, keeping the phenol concentration at 100 mg L^−1^ to avoid phenol depletion throughout the experiment. The peristaltic pump flow was reduced to 16.7 mL min^−1^ to allow for more complex hydrocarbons to form.

An electrochemical procedure (Nova 2.1) consisting of two steps was carried out: first, catalyst conditioning at 0.25 mA cm^−2^ for 30 min; then chronopotentiometry for 5 h. The comparative experiments between Cu foam and Cu/CuO foam were carried out at 60 mA cm^−2^. Additionally, three current density values were selected to test CO_2_RR production of Cu/CuO foam: 40, 60, and 80 mA cm^−2^.

#### 2.3.4 Products Analysis

Gaseous CO_2_RR products were analyzed at 20‐minute intervals by gas chromatography (GC) using a 7890A GC System (Agilent Technologies) equipped with both Flame Ionization Detector (FID) and Thermal Conductivity Detector (TCD). Helium (99.999%, Nippon Gases) was used as the carrier gas. An injection volume of 500 µL and a constant flow rate of 21 mL min^−1^ were selected. Hydrocarbons and CO were separated using a HP‐Plot Q column (30 m × 0.53 mm × 40.0 µm, Agilent Technologies) and quantified via the FID at 250°C (equipped with a methanizer for CO detection). Permanent gases (CO_2_, H_2_, N_2_, and O_2_) were separated on an HP‐Molesieve column (30 m × 0.53 mm × 50.0 µm, Agilent Technologies) and monitored by the TCD at 250°C. Liquid samples from the catholyte and anolyte were collected at the same time intervals. The phenol oxidation was monitored by UV‐vis spectrophotometry, and its products were identified and quantified via GC/MS with a 7820A GC System coupled to a 5977B MSD (Agilent Technologies). For the GC/MS analysis, anolyte samples were prepared as follows: (1) 2 mL aliquots of the aqueous anolytes were transferred to glass vials. To each sample, 39 µL of a cyclohexanol (98%, Sigma‐Aldrich) aqueous solution (5122.26 mg L^−1^) was added as an internal standard (IS) to account for extraction efficiency and volumetric losses, resulting in a final IS concentration of 100 mg L^−1^. (2) Liquid‐liquid extraction was performed by adding 1 mL of ethyl acetate to each solution. The vials were shaken vigorously, and the phases were then allowed to stand until phase separation was complete. 0.5 mL aliquots of the upper organic phases were collected and transferred to GC vials. (3) An additional 0.5 mL of ethyl acetate was added to each GC vial to reach a final volume of 1 mL prior to injection.

Chromatographic separation was achieved using a HP‐5MS UI capillary column (30 × 0.320 mm × 0.25 µm, Agilent Technologies). Helium (99.999%, Nippon gases) was used as the carrier gas at a constant flow rate of 1.0 mL min^−1^. A 5 µL sample was injected in split mode (30:1) with an inlet temperature of 250°C.

The GC temperature program consisted in the following steps: (first) Initial GC oven temperature was held at 85°C for 3 min. (second) Temperature increment to 100°C at a rate of 10°C min^−1^. (third) Temperature increment to 150°C at a rate of 15°C min^−1^. (fourth) Temperature increment to 250°C at a rate of 30°C min^−1^, then held for 1 min. (fifth) Post run at 250°C for 5 min.

The MS source and quadrupole were maintained at 230°C and 150°C, respectively. To protect the filament, a solvent delay of 4.50 min was applied. The mass spectrometer operated in electron ionization (EI, 70 eV) using simultaneous SCAN (m/z 10.00−200.00) and SIM mode. The SIM ions monitored included m/z 100 (cyclohexanol), 94 (phenol), 108 (benzoquinone), and 110 (hydroquinone). Compound identification was performed by comparing mass spectra against the NIST17 library and confirmed using authentic standards.

For the quantification of phenol oxidation products, calibration curves of phenol (99.0%–100.5%, Sigma‐Aldrich), hydroquinone (>99%, Sigma‐Aldrich), and benzoquinone (>99.5% HPLC grade, Sigma‐Aldrich) were built with the following concentrations: 1, 2, 5, 10, 50, and 100 ppm. Quantification was performed using the IS method, where relative response factors were determined from the ratio of the anolyte peak are to the cyclohexanol peak area across the 1–100 ppm calibration range.

The COD of the solution was measured using commercial kits (Merck, ISO 15705) by a Spectroquant Prove 100 VIS spectrophotometer, whereas the phenol concentration was measured by UV/Vis spectroscopy on a Perkin‐Elmer LAMBDA 1050 WB InGaAs UV/Vis spectrophotometer set at λ between 190 and 350 nm. COD removal was calculated according to Equation (S2). COD reduction was used as an indicator of the decrease in oxidizable organic load and not as a direct measurement of carbon mineralization. TOC was not measured in the present study.

## 3 Results and Discussion

### 3.1 Structural and Morphological Investigations

**FIGURE 1:**
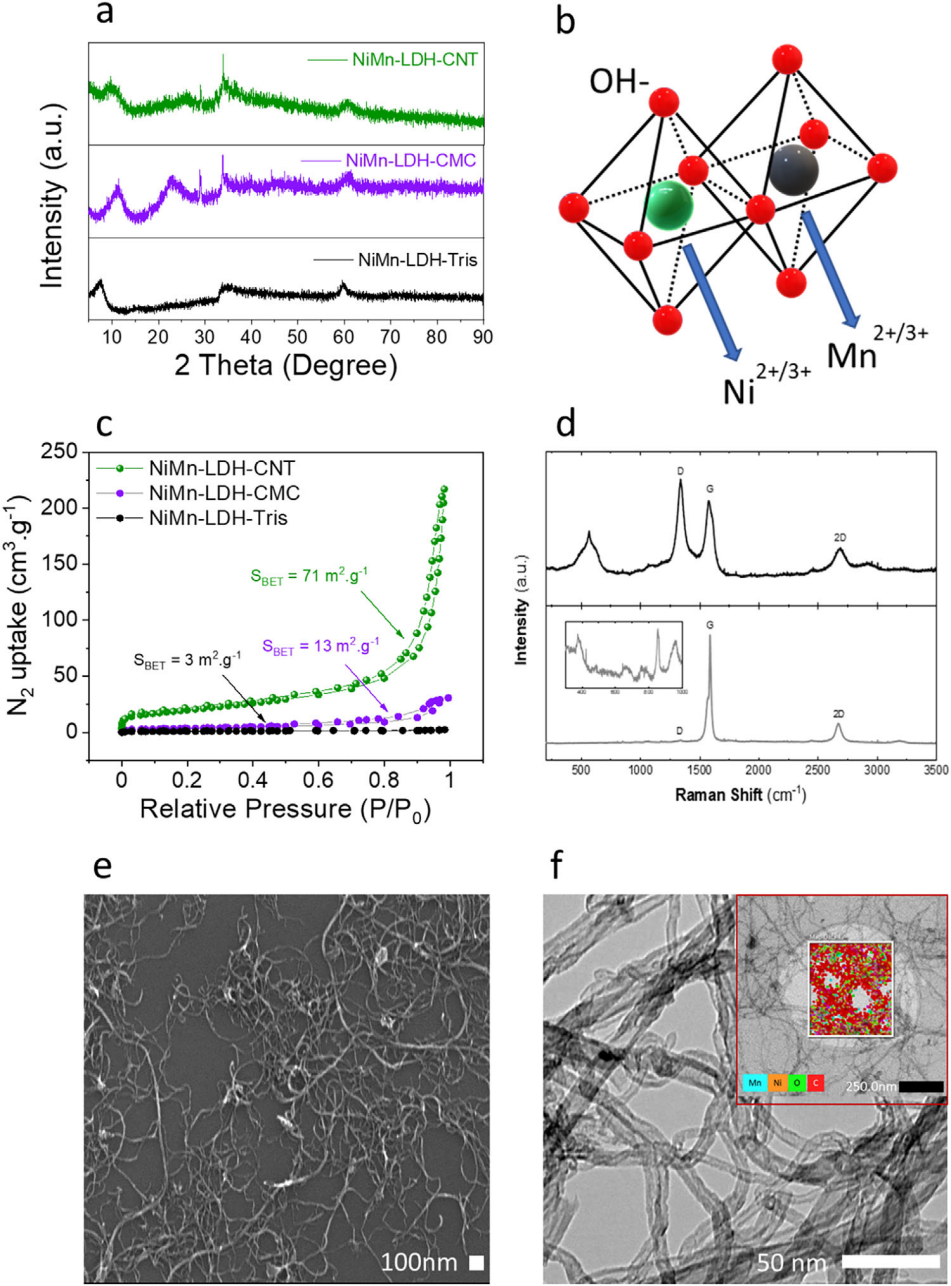
(a) XRD patterns of different NiMn‐LDH samples, showing characteristic peaks corresponding to the layered double hydroxide phase. (b) Brucite‐like structure. (c) Nitrogen adsorption‐desorption isotherms used for BET surface area analysis. (d) Raman spectra of NiMn‐LDH and NiMn‐LDH‐CNT samples, highlighting characteristic vibrational modes associated with hydroxyl groups and metal‐oxygen bonding. (e) SEM image of NiMn‐LDH‐CNT, showing the morphology and distribution of NiMn‐LDH on carbon nanotubes. (f) TEM images of NiMn‐LDH‐CNT, with EDS elemental mapping confirming the distribution of Mn, Ni, O, and C.

Figure 1 presents the structural and morphological features of the three NiMn‐LDH electrode materials, including NiMn‐LDH‐Tris, NiMn‐LDH‐CMC, and NiMn‐LDH‐CNT. XRD patterns (Figure 1a) show the characteristic reflections of a brucite‐like layered double hydroxide phase (Figure 1b). The low‐angle reflections at 2θ ≈ 11–13° and 34–36°, are assigned to the (003) and (006) planes, corresponding to the interlayer spacing (d_BS_). In contrast, the reflection at 2θ ≈ 60° is associated with the lattice parameter *a*, which is associated with the in‐plane metal–metal distance within the brucite‐like layers. The presence of these signals confirms the successful formation of NiMn‐LDH [28, 35]. Analysis of the interlayer spacing shows that NiMn‐LDH‐CNTs and NiMn‐LDH‐CMC exhibit the typical interlayer distance associated with alkoxide synthesis [29, 30], whereas NiMn‐LDH‐Tris displays a lower angle, indicating a larger interlayer spacing due to the covalent functionalization of the LDH with the tris molecule [32]. Subtle variations in peak intensity and broadening among the three samples suggest differences in crystallite size and degree of stacking, which can be attributed to the effects of the different synthesis media or substrates.

BET surface area analysis (Figure 1c) reveals distinctions in textural properties. NiMn‐LDH‐CNT exhibits the highest specific surface area, measured at approximately 71 m^2^ g^−1^, whereas NiMn‐LDH‐CMC and NiMn‐LDH‐Tris show lower values (13 and 3 m^2^ g^−1^, respectively). Such variations likely arise from the porous architecture of carbon nanotubes (enhanced in the pristine CNT compared to the stabilized ones with CMC), which promotes the dispersion of LDH nanosheets and prevents aggregation. The corresponding N_2_ adsorption–desorption isotherms indicate mesoporous characteristics across all samples but with notably enhanced pore volume and surface area in the CNT‐supported material.

Raman spectra (Figure 1d) provide further insight into the bonding environments. The distinct bands around 450–550 cm^−1^ correspond to metal–oxygen vibrations in the LDH lattice, whereas subtle peaks at 1300–1600 cm^−1^ in the CNT‐based sample confirm the presence of carbon (D and G bands). No obvious peaks suggesting secondary phases (e.g., NiO or Mn_3_O_4_) are detected, indicating phase‐pure NiMn‐LDH as the dominant structure.

SEM images and corresponding EDS elemental mappings (Figure S1) illustrate the morphology of each electrode material. NiMn‐LDH‐CNT (Figure 1e and Figure S1a–c) shows a highly interconnected network where thin LDH flakes uniformly anchor onto the CNT framework, consistent with its elevated BET surface area. In contrast, NiMn‐LDH‐CMC (Figure S1d–f) appears more agglomerated, while NiMn‐LDH‐Tris (Figure S1g–i) presents larger, denser particles. The EDS maps reveal homogeneous distributions of Ni, Mn, O, and C for all samples, confirming the uniform incorporation of Mn within the layered hydroxide phase and the successful integration of carbon‐based supports.

Overall, the XRD, BET, Raman, and SEM/EDS analyses confirm the successful synthesis of NiMn‐LDH electrodes under different conditions. Notably, the NiMn‐LDH‐CNT sample exhibits the most pronounced surface area enhancement and a more open, interconnected morphology, features that are expected to be beneficial for electrochemical applications. The differences in microstructure and phase purity among the three samples may ultimately influence their performance in phenol electro‐oxidation, as explored in subsequent sections.

Figure 1f depicts NiMn‐LDH‐CNT, where thin, layered LDH nanosheets are dispersed on a tangled carbon nanotube matrix. The high‐magnification images highlight how the NiMn‐LDH preferentially nucleates and grows along the CNT strands, presumably aided by functional groups introduced during CNT oxidation. The corresponding EDS map (Figure 1f, inset) confirms the uniform presence of Ni, Mn, and O distributed across the carbon framework. Figure S2 compares the nanoscale morphology of NiMn‐LDH prepared under different conditions, as visualized by TEM. In contrast with NiMn‐LDH‐CNT (Figure S2a–c), NiMn‐LDH‐CMC (Figure S2d–f) shows aggregated LDH platelets exhibit flake‐like structures, which appear thicker and less conformal than those on CNT. Although carbon from the carboxymethyl cellulose (CMC) is still present, the LDH particles assemble in larger clusters, potentially reflecting fewer nucleation sites or weaker interfacial interactions compared to the CNT‐supported system. The EDS analysis (Figure S2f, inset) shows Mn and Ni co‐localized with O, confirming the LDH composition even in these more compact regions.

Figure S2g–i presents NiMn‐LDH‐Tris, revealing a denser and more spheroid‐like appearance than its counterparts. These relatively thick aggregates suggest that the Tris‐based synthesis promotes cluster growth rather than the layered or networked architecture observed in the other systems. Elemental mapping (Figure S2i, inset) again confirms the homogeneous spatial distribution of Ni, Mn, and O in the particles.

The TEM images highlight distinct morphological variations among NiMn‐LDH‐CNT, NiMn‐LDH‐CMC, and NiMn‐LDH‐Tris, indicating that the choice of additive or support plays a crucial role in shaping the LDH microstructure. The CNT‐supported system exhibits the most open, extended network of nanosheets, which can, in turn, provide abundant electroactive sites. In contrast, both the CMC and Tris routes yield larger, more aggregated particles, which may limit active surface area and possibly affect their performance in phenol electro‐oxidation, as discussed in subsequent sections.

**FIGURE 2:**
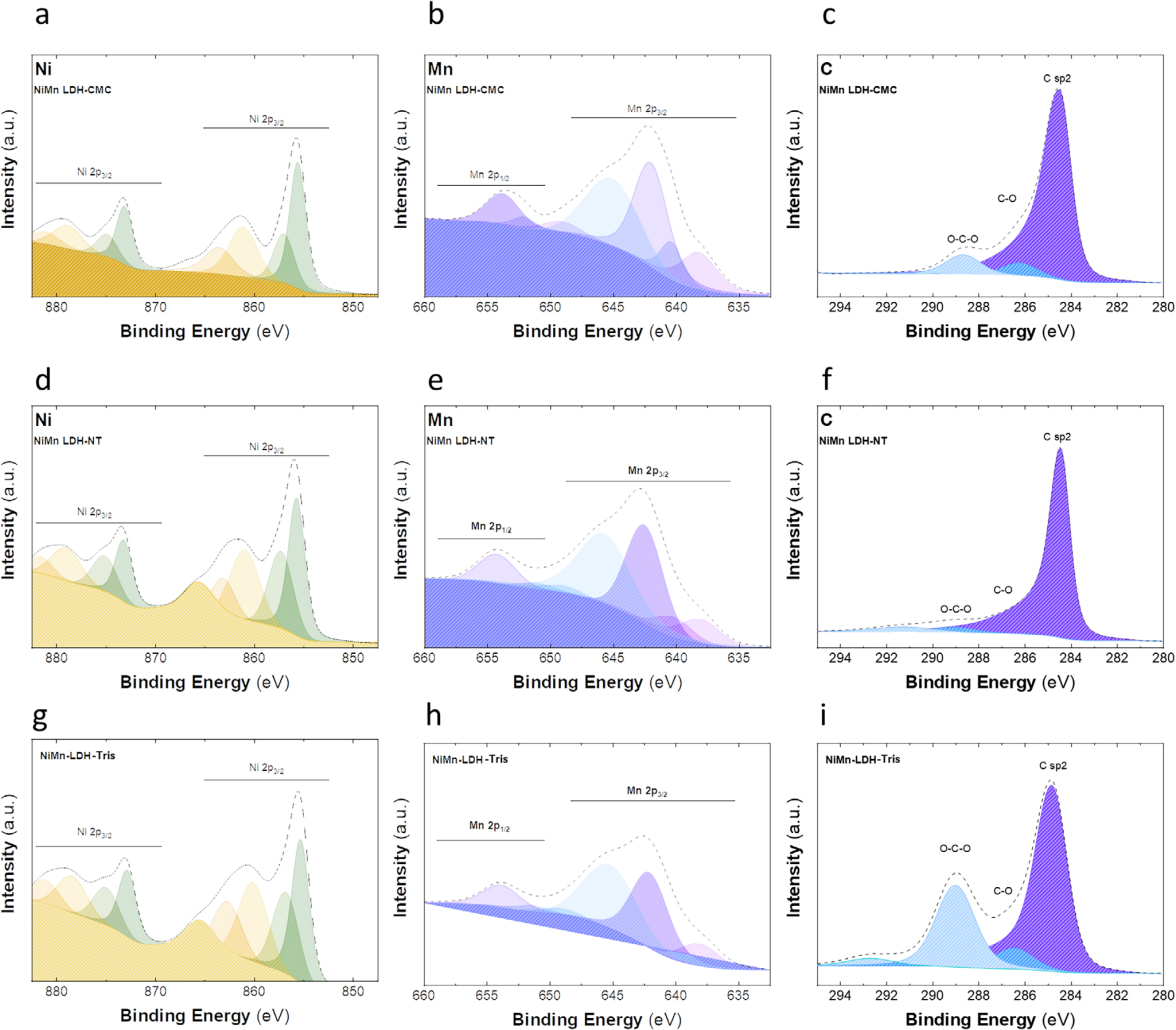
XPS spectra of NiMn‐LDH samples supported on different substrates. (a–c) XPS spectra of NiMn‐LDH‐CNT. (d–f) XPS spectra of NiMn‐LDH‐CMC. (g–i) XPS spectra of NiMn‐LDH‐Tris.

Figure 2 shows the high‐resolution XPS spectra of the three NiMn‐LDH samples. In the Ni 2p_3/2_ spectra (Figure 2a,d,g), the characteristic peak centered around 854–856 eV, along with a satellite at ∼861–864 eV, indicates the coexistence of Ni^2+^ and Ni^3+^ in the LDH framework. For NiMn‐LDH‐CMC (Figure 2a), the Ni 2p peak is relatively broad, suggesting a mix of Ni^2+^ and Ni^3+^ states without a single dominant oxidation state. By contrast, NiMn‐LDH‐CNT (Figure 2d) exhibits a more pronounced high‐binding‐energy shoulder, indicative of an elevated fraction of Ni^3+^. This finding is consistent with the enhanced electron transfer properties of the CNT substrate, which can oxidize Ni^2+^ to Ni^3+^ more effectively. NiMn‐LDH‐Tris (Figure 2g) shows intermediate features, combining a noticeable Ni^2+^ main peak and a smaller Ni^3+^ component, suggesting moderate redox tuning by the Tris‐based synthesis.

NiMn‐LDH‐CMC (Figure 2b) displays Mn 2p peaks around 640–645 eV, reflecting a mixture of Mn^2+^ and Mn^3+^ (possibly Mn^4+^). Meanwhile, NiMn‐LDH‐CNT (Figure 2e) shows a sharper high‐energy peak near 643–644 eV, indicating a higher proportion of oxidized Mn (Mn^3+^ or Mn^4+^). Such enrichment in higher‐valent manganese often correlates with improved redox behavior. NiMn‐LDH‐Tris (Figure 2h) presents broader peaks, suggesting multiple overlapping Mn oxidation states, but with a relatively moderate intensity for the higher‐valent species compared to the CNT‐supported sample.

In NiMn‐LDH‐CMC (Figure 2c), the dominant features at 284.5 eV and 286–288 eV can be attributed to sp^2^ carbon (C‒C) and various oxygenated functionalities (C–O, O–C═O) stemming from the carboxymethyl cellulose. NiMn‐LDH‐CNT (Figure 2f) displays a strong sp^2^‐hybridized carbon peak due to the graphitic nature of CNTs, with a smaller oxygenated shoulder. NiMn‐LDH‐Tris (Figure 2i) also exhibits a main sp^2^ carbon peak but shows moderate contributions from C–O species, possibly resulting from residual Tris‐based moieties or minor adsorbed species.

NiMn‐LDH‐CNT exhibits higher proportions of Ni^3+^ and Mn^3+^/Mn^4+^, which could facilitate enhanced ·OH formation and improved electron transfer for phenol oxidation. By contrast, NiMn‐LDH‐CMC demonstrates a broader distribution of Ni and Mn oxidation states, while NiMn‐LDH‐Tris shows intermediate levels of oxidized metal species.

### 3.2 Electrochemical Performance

**FIGURE 3:**
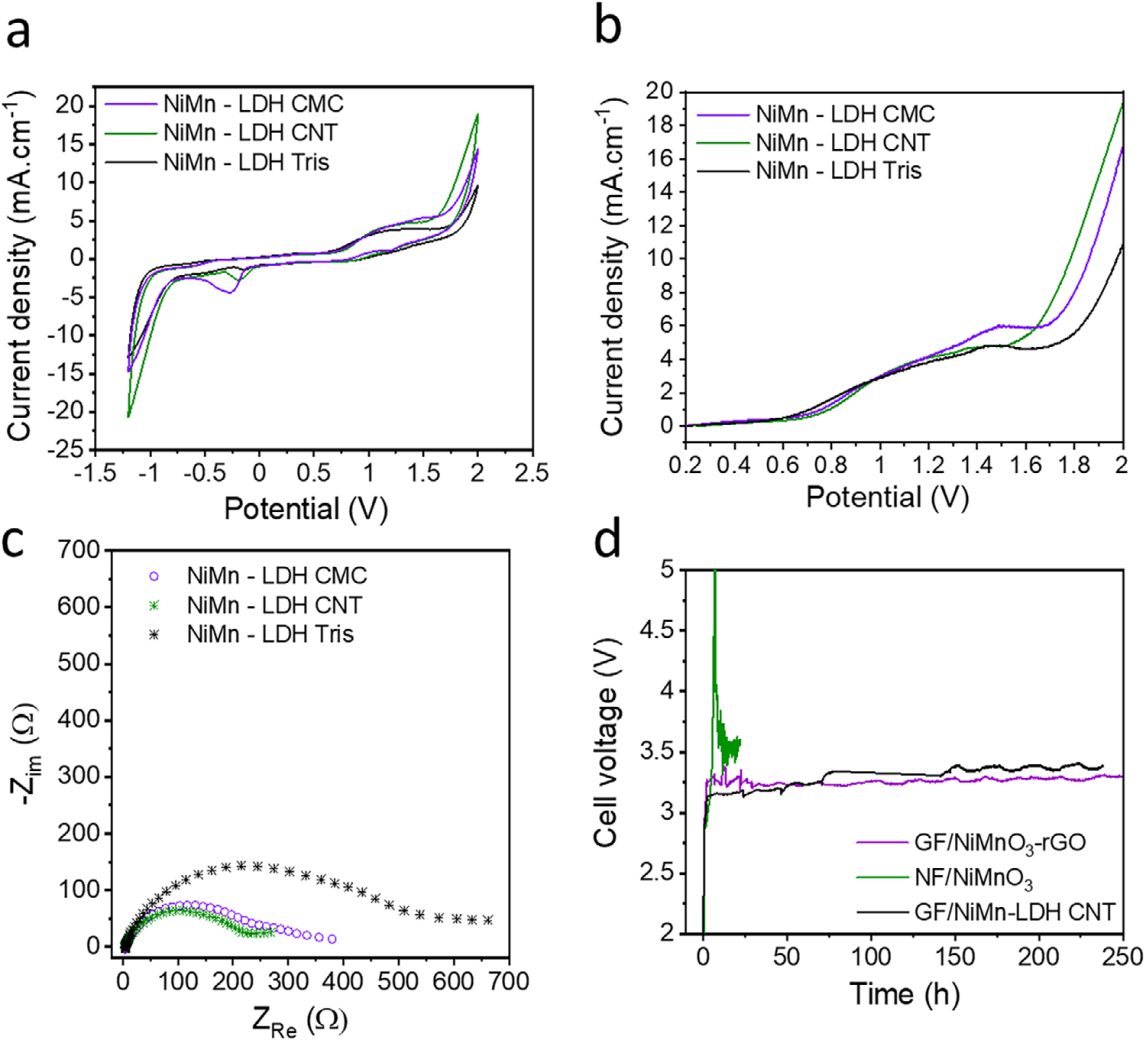
Electrochemical characterization of different NiMn‐LDH electrodes in a flow cell setup using 0.1 M Na_2_SO_4_ electrolyte at neutral pH. (a) CV of NiMn‐LDH electrodes. (b) LSV curves, highlighting the onset potential and catalytic performance of the electrodes for electrochemical oxidation. (c) EIS Nyquist plots, illustrating charge transfer resistance and interfacial properties of the electrodes. (d) Chronopotentiometric profiles comparing the long‐term operational stability of NF/NiMnO_3_, GF/NiMnO_3_–rGO, and GF/NiMn‐LDH‐CNT electrodes. Data for NF/NiMnO_3_ and GF/NiMnO_3_–rGO were obtained from our previous studies.

The electrochemical behavior of the prepared NiMn‐LDH electrodes was evaluated by CV, LSV and EIS to assess their redox activity and catalytic properties. The redox behavior of the catalysts was analyzed by CV in both three‐electrode (Figure S3a–d) and flow cell (Figure 3a) setups. All electrodes display well‐defined anodic and cathodic peaks associated with the reversible Ni^2+^/Ni^3+^ transition, corresponding to the oxidation of Ni(OH)_2_ to NiOOH in alkaline media [12]. This redox couple is widely recognized as the active phase responsible for anodic oxidation reactions. The incorporation of Mn into the LDH structure introduces additional redox flexibility through Mn^2+^/Mn^3+^/Mn^4+^ transitions, which can facilitate proton‐coupled electron transfer processes and promote the formation of reactive oxygen species such as hydroxyl radicals (•OH) [18, 27]. Notably, the NiMn‐LDH‐CNT electrode exhibits higher current densities and sharper redox features, indicating enhanced electrochemical activity and faster charge transfer kinetics compared to the CMC‐ and Tris‐based counterparts.

The catalytic activity toward anodic oxidation was evaluated by LSV in three‐electrode (Figure S3e) and flow cell (Figure 3b) setups. The NiMn‐LDH‐CNT electrode reaches higher current densities at lower applied potentials, demonstrating a more favorable oxidation process and improved catalytic efficiency. In contrast, NiMn‐LDH‐CMC and NiMn‐LDH‐Tris require higher overpotentials to achieve comparable current densities, indicating less efficient catalytic behavior. The superior performance of the CNT‐supported electrode can be attributed to the combined effects of enhanced electrical conductivity, increased electrochemically active surface area, and improved exposure of catalytic sites [20]. The results highlight the critical role of conductive carbon supports in optimizing the electrochemical performance of hybrid NiMn‐LDH catalysts and identify NiMn‐LDH‐CNT as the most promising candidate for phenol electro‐oxidation. Figure 3c presents the EIS Nyquist plots of the different electrodes. The NiMn‐LDH‐CNT electrode exhibits the smallest semicircle diameter among all samples, indicating the lowest charge‐transfer resistance (R_ct_) and thus the most efficient electron transport at the electrode–electrolyte interface. In contrast, NiMn‐LDH‐CMC and NiMn‐LDH‐Tris show larger semicircles, reflecting higher interfacial resistance and slower charge transfer kinetics. The improved conductivity of the CNT‐supported electrode can be attributed to the highly conductive carbon nanotube network, which facilitates electron mobility and enhances electrical connectivity between active sites and the current collector [36, 37]. The electrochemically accessible surface areas were further evaluated by double‐layer‐capacitance measurements (Figure S4). The plots of Δ*j*/2 against scan rate exhibited good linearity, with *R* ^2^ values of 0.9943, 0.9942, and 0.9946 for NiMn‐LDH‐CNT, NiMn‐LDH‐CMC, and NiMn‐LDH‐Tris, respectively. The slopes yielded areal *C*_dl_ values of 29.2, 20.1, and 19.4 mF cm^−2^, respectively. Assuming *C_s_* =  40 µF cm^−2^, the corresponding roughness factors were 730, 502.5, and 485. Considering a projected geometric electrode area of 4.00 cm^2^, the apparent ECSA values were estimated as 2920, 2010, and 1940 cm^2^ for NiMn‐LDH‐CNT, NiMn‐LDH‐CMC, and NiMn‐LDH‐Tris, respectively. Thus, the apparent ECSA of NiMn‐LDH‐CNT was approximately 45% greater than that of NiMn‐LDH‐CMC and 51% greater than that of NiMn‐LDH‐Tris. The larger *C*_dl_ and apparent ECSA of NiMn‐LDH‐CNT are consistent with its higher BET surface area, open nanosheet–CNT architecture, and improved accessibility of the electrode–electrolyte interface. Because a material‐specific *C_s_* value is not available for these heterogeneous NiMn‐LDH composites, the apparent ECSA values are used only for comparative interpretation [38, 39, 40]. Finally, the chronopotentiometric profile presented in Figure 3d shows that GF/NiMn‐LDH‐CNT maintained a nearly constant cell voltage over approximately 200 h of operation, without a pronounced progressive voltage increase. This result demonstrates stable electrochemical operation under the investigated conditions and indicates that no substantial increase in the overall cell resistance occurred during the test. Nevertheless, cell‐voltage stability alone cannot establish whether changes occurred in the crystal structure, morphology, surface oxidation states, elemental composition, or catalyst loading. Because post‐operation structural and compositional characterization was not performed, the present durability result should be interpreted as evidence of operational electrochemical stability rather than definitive proof of structural stability. Future studies should combine extended electrolysis with post‐operation XRD, XPS, SEM/EDS, and analysis of dissolved Ni and Mn to evaluate catalyst reconstruction, detachment, and metal leaching.

### 3.3 Electrochemical Oxidation of Organic Pollutants

**FIGURE 4:**
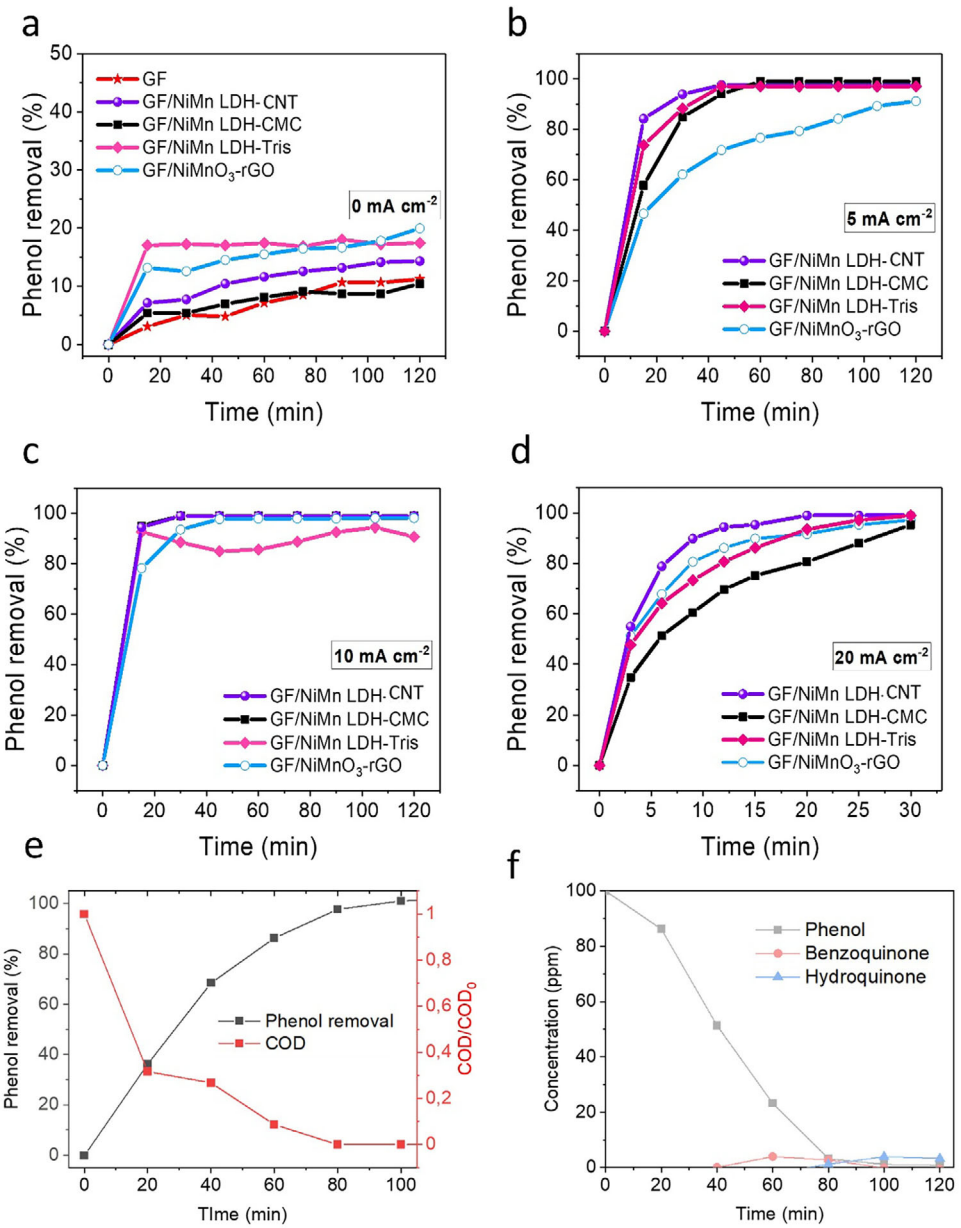
Comparison of phenol removal efficiency for different NiMn‐LDH electrodes at varying current densities and evaluation under synthetic‐wastewater conditions. (a–d) Phenol degradation profiles at applied current densities of 0, 5, 10, and 20 mA cm^−2^, showing the influence of current density on electrochemical oxidation performance. (e‐f) Experiments performed using synthetic water doped with 100 mg L^−1^ phenol as electrolyte.

Figure 4 summarizes the phenol electro‐oxidation performance of different Ni‐based catalysts at various current densities (0–20 mA cm^−2^). Phenol removal profiles for GF/NiFe‐LDH, GF/NiMn‐LDH, and GF/NiMnO_3_–rGO (published in an earlier study by our group [26, 27]) are compared to demonstrate the superior effectiveness of NiMn‐LDH relative to NiFe‐LDH and even to our previously optimized NiMnO_3_–rGO material (Figure S5) [26, 27]. NiMn‐LDH achieves nearly 100% phenol removal within 20–30 min, while NiFe‐LDH requires over 40 min to reach only about 70%–80% removal under identical operating conditions, indicating that Mn incorporation fosters a more rapid, higher‐efficiency degradation pathway [41]. These findings are consistent with recent reports of defect‐promoted Ni–Mn sites, which exhibit a high deprotonation propensity and facilitate faster electron transfer during organic oxidation reactions [18]. A quantitative comparison with representative phenol electro‐oxidation systems is presented in Table 1. A literature survey revealed that NiMn‐LDH materials have rarely been investigated specifically for electrochemical phenol degradation; most studies of NiMn‐LDH electrocatalysts concern OER or the oxidation of biomass‐derived and small organic molecules. Therefore, the comparison includes the closest Ni–Mn‐based phenol‐oxidation electrodes and representative conventional and three‐dimensional electrochemical systems. Because reactor configuration, electrode geometry, electrolyte composition, pH, current density, and treatment time differ substantially among studies, the table is intended to provide contextual benchmarking rather than a strict ranking of intrinsic catalytic activity.

Under the investigated conditions, GF/NiMn‐LDH‐CNT achieved more than 95% phenol removal within approximately 10–15 min at 10–20 mA cm^−2^ and nearly complete removal within approximately 40 min at 5 mA cm^−2^. This treatment time is shorter than that observed for the NiFe‐LDH control and the previously reported NiMnO_3_ electrode. It is also shorter than the 180 min required by the SnO_2_–Sb_2_O_3_/GAC three‐dimensional system to achieve 99.65% phenol removal, although that study used a batch reactor operated at pH 3 and a fixed cell voltage of 15 V [42]. The comparison therefore supports the favorable degradation kinetics of NiMn‐LDH‐CNT under near‐neutral conditions. Nevertheless, differences in reactor architecture, electrode surface area, hydrodynamics, and electrical input prevent the removal percentages from being interpreted as a direct comparison of intrinsic catalyst activity. Future benchmarking should include normalized kinetic constants, current efficiency, and energy consumption under standardized operating conditions.

Having validated NiMn‐LDH as a superior alternative to NiFe‐LDH, we then investigated the three different NiMn‐LDH variants, NiMn‐LDH‐Tris, NiMn‐LDH‐CMC, and NiMn‐LDH‐CNT at current densities of 0, 5, 10, and 20 mA cm^−2^, as shown in panels (Figure 4a–d). At 0 mA cm^−2^, which represents physico‐chemical adsorption (Figure 4a), minimal removal occurs (<20%) primarily due to limited electrochemical driving force. High adsorption behavior was observed in the adsorption profile of NiMn‐LDH‐Tris, similar to what has been reported for NiFe‐LDH functionalized with triethoxysilanes and an amine, which was used for the removal of Cr(VI) [43]. Under 5 mA cm^−2^ (Figure 4b), GF/NiMn‐LDH‐CNT demonstrates a steep initial removal rate, reaching nearly 100% within 40 min, whereas NiMn‐LDH‐Tris and NiMn‐LDH‐CMC each require an additional 20–30 min to approach similar removal levels. This difference is in line with the enhanced electron conductivity and deprotonation capability observed in CNT‐supported electrodes, which promote hydroxyl radical (•OH) generation and a higher turnover of phenol oxidation intermediates [36].

At 10 and 20 mA cm^−2^ (Figure 4c,d), all NiMn‐LDH variants exhibit improved phenol removal due to the higher applied bias, which accelerates the Ni/Mn centers activity. Notably, GF/NiMn‐LDH‐CNT consistently achieves >95% removal within 10–15 min, outperforming both GF/NiMn‐LDH‐Tris and GF/NiMn‐LDH‐CMC. This behavior is consistent with the combined effects of the more open dispersion of NiMn‐LDH nanosheets, the higher accessible surface area, and the lower interfacial charge‐transfer resistance provided by the conductive CNT scaffold, yielding better dispersions of active sites and lower charge‐transfer resistance, thus aligning with the EIS findings (Figure 3c) [36]. By contrast, NiMn‐LDH‐CMC, despite showing relatively high removal efficiency at extended times, suffers from partial aggregation and moderately slower reaction kinetics due to less efficient electron conduction.

The results demonstrate that NiMn‐LDH outperforms NiFe‐LDH under the investigated phenol electro‐oxidation conditions, confirming the beneficial synergy between Ni and Mn for generating reactive oxygen intermediates while mitigating competing side reactions [26, 27, 41]. Among the three hybrid NiMn‐LDH formulations, the CNT‐supported system offers superior kinetics, selectivity, and long‐term stability due to enhanced deprotonation propensity, better electron transport, and more effectively exposed active sites. These insights align well with previous studies on nickel‐based layered hydroxides for organic oxidation, reinforcing the importance of catalyst design for achieving rapid and robust electrochemical wastewater treatment.

In addition to the effect of the current density on phenol removal, further experiments evaluating the influence of electrolyte conductivity on the electrochemical oxidation process were performed by varying the concentration of Na_2_SO_4_ (from 0.1 to 0.001 M) used as the supporting electrolyte (Figure S6). As shown in Figure S6a,b, no significant differences (<5%) in COD removal and phenol removal efficiency were observed when using 0.1 M and 0.01 M Na_2_SO_4_, indicating that both concentrations provide sufficient ionic conductivity to sustain efficient charge transport within the electrochemical cell. However, when the Na_2_SO_4_ concentration was further reduced to 0.001 M, a noticeable reduction in phenol removal kinetics was observed. This result was further confirmed by the COD removal reaching 50% after 60 min while more than 70% was achieved when using higher concentrated electrolyte. This behavior suggests that at such low electrolyte concentrations the ionic conductivity of the solution becomes insufficient, resulting in increased ohmic resistance and reduced electrochemical efficiency. Still, control experiments performed with bare graphite felt (GF) electrodes showed a significant impact on phenol removal when the LDH electrocatalyst was incorporated into the substrate (Figure S6c). This interpretation is further supported by the chronopotentiometry profiles shown in Figure S6d,e, where the cell voltage increases as the Na_2_SO_4_ concentration decreases for both NiMn‐LDH‐CNT/GF and bare GF electrodes. The increase in cell potential reflects the higher internal resistance of the system under low electrolyte concentrations, which leads to less efficient current distribution and ultimately reduces the oxidation performance.

To further assess performance under more realistic conditions, electrochemical experiments were conducted using NiMn‐LDH‐CNT in a synthetic water matrix (composition detailed in Table S1). Phenol degradation and chemical oxygen demand (COD) removal were initially monitored by UV‐vis spectrophotometry. As shown in Figure 4e, complete removal of both phenol and COD was achieved within 120 min. Phenol and COD degradation proceeded rapidly during the initial 20 min, eventually converging both removal trends. The substantial COD decrease indicates extensive oxidation of phenol and other oxidizable constituents. However, COD removal does not directly quantify carbon removal, since partially oxidized organic compounds can remain in solution with a lower oxygen demand. As TOC was not measured, the extent of mineralization cannot be established from the present results. Given the inherent limitations of UV‐vis analysis in complex matrices containing additional organic species (e.g., urea, peptone, and meat extract), complementary GC–MS analyses were performed. Time‐resolved GC–MS measurements (Figure 4f and Table S2) confirmed the progressive depletion of phenol, with complete removal observed after ∼80 min, alongside the formation of low concentrations (<10 ppm) of intermediate products such as benzoquinone and hydroquinone. Overall, these results demonstrate the robustness and effectiveness of the NiMn‐LDH‐CNT electrode for phenol degradation under realistic wastewater conditions.

### 3.4 Hybrid Electrochemical Configuration: CO_2_ Reduction Coupled to Phenol Oxidation

**FIGURE 5:**
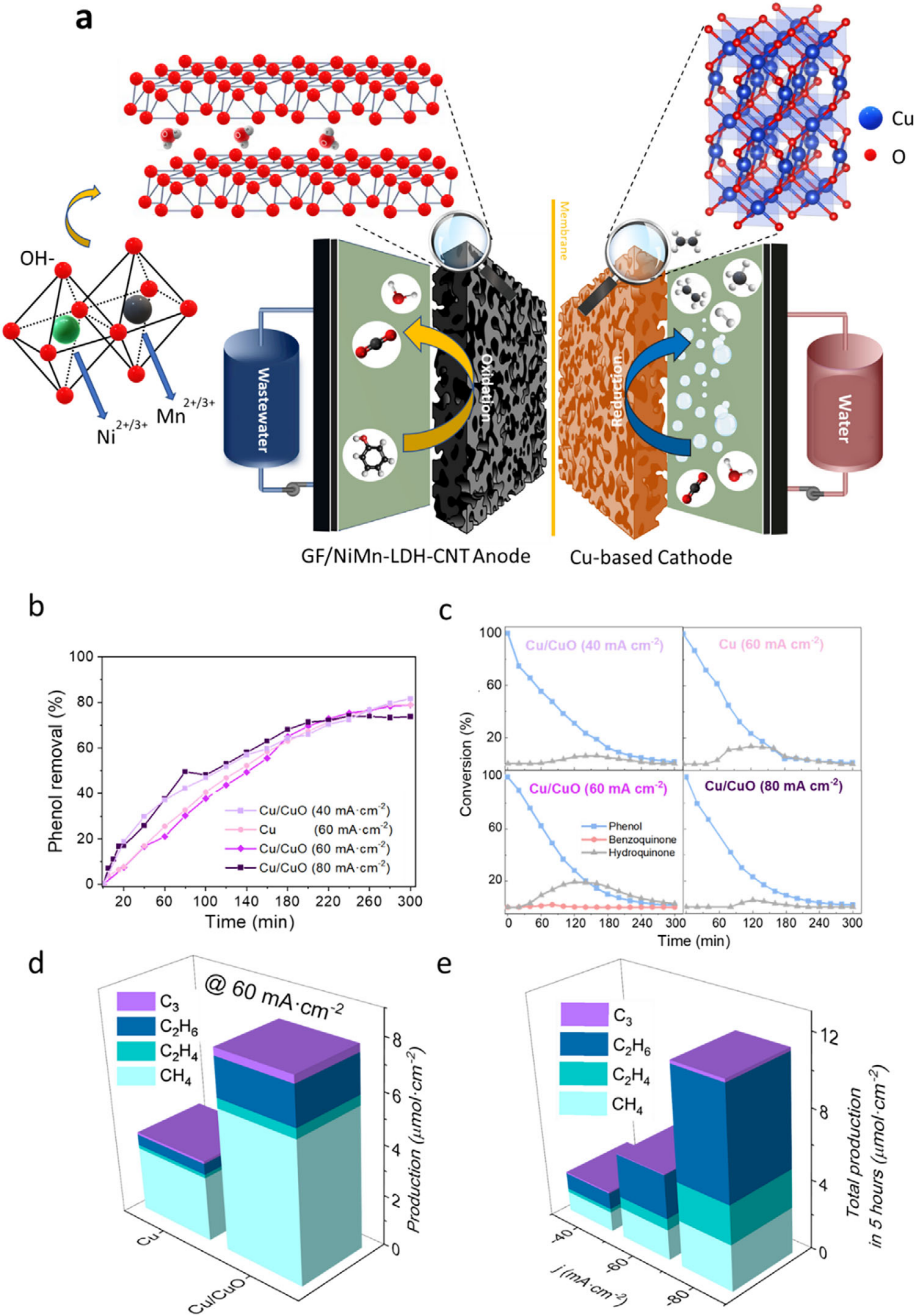
(a) Scheme of the two‐compartment electrochemical cell. (b) Phenol removal in the anodic compartment at different current densities (40, 60, and 80 mA cm^−2^). The experiment was performed using 1 L solution of 100 mg L^−1^ phenol. (c) GC‐MS analysis showing the oxidation of phenol to hydroquinone and benzoquinone at 40, 60, and 80 mA cm^−2^. d) Comparative accumulated hydrocarbons production at 60 mA cm^−2^ of bare commercial Cu and Cu/CuO cathodes during 5 h. (e) Accumulated hydrocarbons production of Cu/CuO cathode 40, 60, and 80 mA cm^−2^ during 5 h.

Paired electrolysis offers an opportunity to use both electrode reactions productively by replacing conventional anodic OER with the oxidation of an organic substrate. Previous studies have demonstrated this concept by coupling phenol oxidation with hydrogen production and by integrating methanol electro‐oxidation with cathodic hydrogen generation. In the present work, this strategy is extended to the simultaneous remediation of phenol‐containing water and conversion of CO_2_. Figure 5a illustrates the two‐compartment electrochemical cell employed, in which phenol oxidation occurs at the NiMn‐LDH‐CNT anode while CO_2_ reduction proceeds at the Cu‐based cathode. Unlike systems in which the anodic organic compound is primarily treated as a chemical feedstock, the present configuration uses phenol as a model wastewater contaminant. The configuration therefore demonstrates the feasibility of combining pollutant degradation with cathodic carbon utilization. From a process‐integration perspective, coupling phenol oxidation with CO_2_RR allows the same electron flow to support two desired transformations. If operated independently, CO_2_RR would require an auxiliary anodic reaction, conventionally OER, while electrochemical phenol oxidation would require a separate cathodic reaction. Combining the two target half‐reactions could therefore reduce the number of independently operated electrochemical processes and avoid allocating electrical input to non‐target counter‐electrode reactions. In principle, organic oxidation may also proceed at a lower anodic potential than OER, potentially decreasing the full‐cell voltage required for CO_2_ conversion. Nevertheless, this energetic benefit depends on the reaction kinetics, electrode overpotentials, ohmic losses, product selectivity, and current efficiencies of the complete system. The present experiments demonstrate simultaneous phenol degradation and CO_2_ conversion, but they do not provide a direct quantitative comparison with conventional CO_2_RR coupled to OER. Accordingly, an energy advantage cannot be established from the available data. Such an assessment would require measurements of the full‐cell voltage for CO_2_RR–phenol oxidation and CO_2_RR–OER at identical current densities and operating conditions, followed by calculation of the electrical‐energy input relative to phenol removed and cathodic product generated. The present hybrid configuration should therefore be considered a proof of concept for process integration rather than a demonstration of reduced energy consumption.

To further quantify the reaction products and intermediates, anolyte identification and quantification were performed by GC/MS using the internal standard method. Calibration curves were constructed in SCAN mode for phenol (m/z 94) and in SIM mode for hydroquinone and benzoquinone (m/z 110 and 108, respectively), using cyclohexanol (m/z 100) as the internal standard (Figure S7). Due to the chemical instability of quinones in calibration solutions, their respective calibration curves were fitted using the combined signal of m/z 108 and 110 at each retention time. All calibrations showed excellent linearity (R^2^> 0.998), confirming the reliability of the extraction and analytical protocol.

The analysis of the electrolysis experiments revealed a continuous decrease in phenol concentration accompanied by the formation of aromatic intermediates (Figure 5c). Hydroquinone was consistently identified as the dominant intermediate under all operating conditions, indicating that phenol oxidation proceeds mainly through hydroxylation pathways. In contrast, benzoquinone was detected only in trace quantities, specifically during the experiment performed at 60 mA cm^−2^ using the Cu/CuO cathode. This observation suggests that benzoquinone is either rapidly reduced or further oxidized under the electrochemical conditions. Furthermore, the carbon mass balance could not be fully accounted for by the aromatic species quantified by GC–MS. This result suggests the formation of additional products outside the scope of the applied analytical method, potentially including low‐molecular‐weight organic acids. However, because neither these compounds nor TOC and inorganic carbon were quantified, the unaccounted carbon cannot be assigned to mineralization as CO_2_.

The performance of the cathodic compartment was evaluated using copper foam and Cu/CuO foam electrodes. SEM analysis (Figure S8) shows that the macroscopic porous structure of the copper foam remains intact after calcination, preserving its intrinsic porosity (95%–98%). However, high‐magnification images reveal the formation of a dense network of CuO nanowires (approximately 10 µm in length and 100 nm in diameter). This hierarchical nanostructure significantly increases the electrochemically active surface area and provides a three‐dimensional scaffold that enhances the density of catalytic sites available for CO_2_ reduction [44].

These morphological features directly influence the electrocatalytic performance toward both the hydrogen evolution reaction and CO_2_RR. Using pristine Cu foam as a reference, the Cu/CuO foam exhibited a substantial enhancement in activity, reaching an approximately 8.7‐fold increase at 60 mA cm^−2^ (Figure S9a). The gaseous products obtained at 60 mA cm^−2^ are summarized in Figure 5d. Compared with bare Cu foam, the Cu/CuO electrode shows a noticeable increase in hydrocarbon formation, with an overall 2.7‐fold enhancement in the production of CH_4_, C_2_H_4_, C_2_H_6_, and C_3_ species. While part of this improvement can be attributed to the increased electrochemically active surface area of the nanowire network, the enhanced catalytic activity likely also arises from electronic effects induced by the presence of CuO. In particular, partially oxidized Cu^δ+^ species (0< δ < 2), defect‐rich sites, and Cu^0^/Cu^δ+^ interfacial domains are known to modify adsorption energetics and influence reaction pathways in CO_2_RR [45].

Importantly, the appearance of significant CO production on the Cu/CuO foam (1.6 mmol cm^−2^), which was not detected on pristine Cu (Figure S9b), suggests that Cu^δ+^ species and coordinatively unsaturated surface sites facilitate the stabilization of *CO intermediates [46]. Such oxidized or defect‐rich domains have been widely associated with modified adsorption energetics and increased *CO coverage during CO_2_RR. Although higher *CO coverage may promote C–C coupling toward C_2+_ products, the simultaneously enhanced HER activity of the Cu/CuO foam likely increases the local surface concentration of adsorbed hydrogen (H_ads_). Elevated H_ads_ coverage can accelerate hydrogenation pathways, competing with C–C coupling reactions and favoring methane formation [47].

To further evaluate catalytic behavior, the Cu/CuO foam electrode was tested at three different current densities (40, 60, and 80 mA cm^−2^), as shown in Figure 5e. A pronounced non‐linear increase in total product formation, particularly hydrogen, was observed when increasing the current density from 40 to 60 mA cm^−2^ (Figure S10a). This sharp increase suggests that at higher overpotentials, the Cu/CuO nanowire structure activates additional coordinatively unsaturated catalytic sites that remain largely inactive at lower potentials.

In contrast, CO production showed a nearly linear increase with increasing current density (Figure S10b), indicating that the two‐electron reduction of CO_2_ to *CO is favored at higher overpotentials. The presence of Cu^δ+^ species and surface defects likely facilitates *CO stabilization and its subsequent desorption or further reduction [48].

Interestingly, the highest selectivity toward ethane was observed at the lowest tested current density (40 mA cm^−2^). Under these moderate overpotential conditions, the hierarchical nanowire surface likely retains a higher fraction of Cu^δ+^ species, which stabilize *CO intermediates while maintaining a relatively low surface coverage of H_ads_, thereby favoring C–C coupling pathways [49]. When the current density increases to 60 mA cm^−2^, the reaction environment becomes dominated by hydrogenation reactions, resulting in enhanced methane production. However, at 80 mA cm^−2^, the increased flux of carbonaceous intermediates appears to overcome the kinetic limitations of C–C coupling, leading to a renewed increase in C_2+_ product formation [50] (Figure 5e).

## 4 Conclusion

In this work, hybrid NiMn‐LDH electrodes were investigated as active anodic materials for the electrochemical oxidation of phenol in aqueous systems. The electrochemical characterization confirmed that the incorporation of different carbon‐based modifiers significantly influences the catalytic behavior of the hybrid NiMn‐LDH materials. In particular, the NiMn‐LDH‐CNT electrode exhibited the highest electrochemical activity, which can be attributed to the improved electrical conductivity and enhanced accessibility of catalytic sites provided by the conductive carbon nanotube network. The CNT‐supported electrode also maintained a nearly constant cell voltage over approximately 200 h, demonstrating long‐term operational stability under the investigated conditions. However, because post‐operation structural and compositional characterization was not performed, preservation of the LDH structure, surface composition, and catalyst loading after prolonged electrolysis remains to be confirmed.

The catalytic performance of the materials was evaluated through phenol electro‐oxidation experiments conducted at different current densities. All electrodes demonstrated efficient phenol degradation, with the reaction rate increasing with the applied current density. Product analysis revealed that hydroquinone was the main aromatic intermediate formed during phenol oxidation, while benzoquinone was detected only in trace amounts, suggesting that it undergoes rapid further transformation under the applied electrochemical conditions. The decrease in COD, together with the low concentrations of detected aromatic intermediates, indicates that phenol underwent further oxidation beyond its initial transformation. Nevertheless, the absence of TOC and inorganic‐carbon measurements prevents quantification of mineralization, and the unaccounted carbon may include low‐molecular‐weight products outside the scope of the applied GC–MS method. Beyond conventional electro‐oxidation experiments, the optimized NiMn‐LDH anode was successfully integrated into a hybrid electrochemical system coupling phenol oxidation with the CO_2_ reduction reaction (CO_2_RR). In this configuration, the anodic degradation of phenol was combined with the cathodic conversion of CO_2_ using Cu‐based foam electrodes. The Cu/CuO foam cathode showed significantly enhanced catalytic activity compared to pristine Cu foam, which was attributed to the formation of a hierarchical nanowire structure and the presence of partially oxidized Cu^δ+^ species that modify the electronic structure of the catalytic surface. These features promoted the stabilization of key reaction intermediates and resulted in improved hydrocarbon formation, including CH_4_, C_2_H_4_, C_2_H_6_, and C_3_ products. The hybrid configuration demonstrates the feasibility of integrating NiMn‐LDH‐mediated phenol oxidation with CO_2_ reduction at a Cu/CuO cathode, allowing wastewater treatment and carbon conversion to proceed simultaneously within a single electrochemical circuit. This integration avoids the need to pair each target reaction with a separate non‐target counter‐electrode process and may offer a route toward improved process efficiency. However, because a directly comparable CO_2_RR–OER control was not evaluated, no quantitative energy advantage can be concluded from the present results. Future work should compare full‐cell voltages, Faradaic efficiencies, carbon balances, and specific energy consumption under matched operating conditions.

## Conflicts of Interest

The authors declare no conflicts of interest.

## Supporting information




**Supporting File**: gch270155‐sup‐0001‐SuppMat.docx.

## Data Availability

The data that support the findings of this study are available on request from the corresponding author. The data are not publicly available due to privacy or ethical restrictions.
