# Supplementary material for: Hybrid NiMn‐LDHs as Active Materials for Electro‐Oxidation of Organic Pollutants
Source: Glob Chall. 2026 Sep 25;10(9):e70155. doi: 10.1002/gch2.70155 (PMC13613091; doi:10.1002/gch2.70155)
Supplement: Supplementary file 1 [file GCH2-10-e70155-s001.docx]

Supporting Information

Hybrid NiMn-LDHs as Active Materials for Electro-Oxidation of Organic Pollutants

Keyvan Mirehbar ^[a,e]^, Javier Llorente-López ^[b]^, Alvaro Seijas-Da Silva^[c,d]^, Camilo Jaramillo-Hernández ^[c]^, Petra Batinić ^[a]^, Paula Porawski^[a]^, Miguel García-Tecedor^[b]^, Víctor A. de la Peña O’Shea ^[b]^, Jesús Palma ^[a]^, Gonzalo Abellán*^[c]^, Julio J. Lado*^[a]^

**Structural Characterization**

**Figure S1.** (a–c) SEM images of NiMn-LDH-CNT at different magnifications, showing the morphology and distribution of NiMn-LDH on carbon nanotubes, with (c) displaying EDS elemental mapping. (d–f) SEM images of NiMn-LDH-CMC at different magnifications, with (f) showing EDS elemental mapping. (g–i) SEM images of NiMn-LDH-Tris, with (i) presenting EDS elemental mapping of Ni, Mn, O, and C distribution.

**Figure S2.** TEM images of NiMn-LDH deposited on different carbons. (a–c) TEM images of NiMn-LDH-CNT at increasing magnifications, with (c) EDS elemental mapping confirming the distribution of Mn, Ni, O, and C. (d–f) TEM images of NiMn-LDH-CMC, with (f) showing EDS mapping confirming the elemental composition. (g–i) TEM images of NiMn-LDH-Tris with (i) presenting EDS elemental mapping of Ni, Mn, O, and C.

**Electrochemical Characterization**

**Figure S3.** Electrochemical characterization of different NiMn-LDH electrodes in three-electrode setup using 0.1 M Na_2_SO_4_ electrolyte at neutral pH. (a) CV performed at 20 mV s^-1^. CV´s performed at different scan rates between 1-100 mV s^-1^ for (b) LDH-CNT; (c) LDH-CMC; (d) LDH-Tris. (e) LSV performed at 1 mV s^-1^.

**Figure S4.** Double-layer-capacitance analysis of the different NiMn-LDH electrodes in the flow-cell configuration using 0.1 M Na_2_SO_4_. Cyclic voltammograms recorded within material-specific non-Faradaic potential windows at scan rates of 20, 50, 100, and 200 mV s^-1^ for (a) NiMn-LDH-CNT, (b) NiMn-LDH-CMC, and (c) NiMn-LDH-Tris. (d) Linear plots of $\Delta j/2$ versus scan rate used to determine the areal double-layer capacitance.

**Evaluation of Phenol Degradation**

The removal efficiencies of phenol and COD were calculated by **Equations S1 and Equation S2**:

Phenol removal efficiency (in %) =$\frac{C_{\text{0}} - C_{\text{t}}}{C_{\text{0}}} \times100$	**Equation S1**

COD removal efficiency (in %) = $\frac{\mathrm{COD}_{\text{0}} - \mathrm{COD}_{\text{t}}}{\mathrm{COD}_{\text{0}}}$ $\times100$	**Equation S2**

where *C*_0_ and *C*_t_ are the initial and final concentrations of phenol, respectively, while COD_0_ and COD_t_ are the initial and final COD (in mg O_2_ L^-1^) of the solution.

A pseudo-first-order kinetic equation was used to fit the data of the phenol degradation, according to **Equation S3**:

ln$\frac{C_{\text{0}}}{C_{\text{t}}}$= *kt*	**Equation S3**

where *k* is the reaction rate constant.

**Figure S5.** Phenol degradation profile of GF/NiMn-LDH electrode comparing GF/NiFe-LDH.

**Figure S6.** Effect of the supporting electrolyte concentration on phenol electro-oxidation performance. (a) COD removal profiles using the GF/NiMn-LDH-CNT electrode at different Na_2_SO_4_ concentrations. (b) Phenol removal profiles using the GF/NiMn-LDH-CNT electrode at different Na_2_SO_4_ concentrations. (c) Control experiments using bare graphite felt (GF) under the same conditions. Chronopotentiometric responses for (d) GF/NiMn-LDH-CNT and (e) bare GF electrodes.

**Table S1.** Synthetic water was prepared by dissolving the specified components in 1 L of tap water (conductivity: 271 ± 49; pH = 7.69 ± 0.55), to better approximate realistic water matrices.

|  | **Concentration** (mg L^-1^) |
| --- | --- |
| Peptone | 160 |
| Meat extract | 110 |
| Urea | 30 |
| K_2_HPO_4_ | 28 |
| NaCl | 50 |
| CaCl_2_·2H_2_O | 4 |
| MgSO_4_·7H_2_O | 2 |
| Phenol | 100 |
| Conductivity | 394 |
| pH | 7.51 |

**Hybrid Electrochemical Configuration**

**Figure S7.** Calibration curves of the studied analytes for the GC-MS measurements.

**Figure S8**. Morphology of the employed Cu-based cathodes. (a) Optical image of the 2x2 cm bare commercial Cu foam cathode. (b) FE-SEM image of the bare Cu cathode. (c) Optical image of the 2x2 cm Cu/CuO cathode. (d) FE-SEM image of the Cu/CuO cathode. (e) and (f) Magnifications of the CuO nanowires.

**Table S2.** Parameters of the calibration curves.

| **Analyte** | **Slope** | **y-intercept** | **R^2^** |
| --- | --- | --- | --- |
| Phenol | 0.8016 | -0.0575 | 0.9995 |
| Benzoquinone | 0.2026 | -0.203 | 0.9994 |
| Hydroquinone | 0.5156 | -0.709 | 0.998 |

**Figure S9**. Measured gas products in the cathodic compartment using the Cu and Cu/CuO electrodes at 60 mA cm^-2^. (a) Total production. (b) CO production.

**Figure S10**. Measured gas products in the cathodic compartment using Cu/CuO electrode at 40, 60 and 80 cm^-2^. a) Total production. b) CO production.
